# Comparison of hospital worker anxiety in COVID-19 treating and non-treating hospitals in the same city during the COVID-19 pandemic

**DOI:** 10.1186/s13584-020-00413-1

**Published:** 2020-10-21

**Authors:** Yael Milgrom, Yuval Tal, Aharon S. Finestone

**Affiliations:** 1 The Liver Unit, Internal Medicine, Hadassah University Hospital, Ein Kerem, 911200 Jerusalem, Israel; 2 Occupational Health Unit, Internal Medicine, Hadassah University Hospital, Ein Kerem, Jerusalem, Israel; 3 Department of Orthopaedics, Shamir Medical Center, Zerifin and Faculty of Medicine, Tel Aviv University, Tel Aviv, Israel

**Keywords:** Anxiety, COVID-19 pandemic, Questionnaire, Risk factors, Lock-down, Hospital workers

## Abstract

**Background:**

The Hadassah Medical Organization operates two hospitals in Jerusalem. During the COVID-19 pandemic it made an administrative decision to operate one hospital as a COVID-19 treatment hospital (CTH) and to have the second function as a non-COVID-19 treating hospital (NCTH) offering general medical services. The purpose of this study was to assess how this decision affected hospital worker anxiety.

**Methods:**

From April 27 to May 1, during the COVID-19 pandemic in Israel, while the country was under lock-down, an electronic questionnaire survey was carried out among hospital workers of the CTH and NCTH. The questionnaire includes personal demographics and attitudes about COVID-19 and assesses present anxiety state using the State-Trait Anxiety Inventory for Adults (STAI-S) validated questionnaire. A STAI-S score of ≥45 was considered to represent clinical anxiety.

**Results:**

Completed questionnaires were received from 1570 hospital employees (24%). 33.5% of responders had STAI-S scores ≥45. Multivariable regression analysis showed that being a resident doctor (odds ration [OR] 2.13; 95% CL, 1.41–3.23; *P* = 0.0003), age ≤ 50 (OR, 2.08; 95% Cl, 1.62–2.67; *P* < .0001), being a nurse (OR, 1.29; 95% CL, 1.01–1.64; *P* = 0.039), female gender (OR, 1.63; 95% CL, 1.25–2.13; *P* = 0.0003) and having risk factors for COVID-19 (OR, 1.51; 95% CL, 1.19–1.91; *P* = 0.0007), but not hospital workplace (*p* = 0.08), were associated with the presence of clinical anxiety. 69% of the responders had been tested for COVID-19, but only nine were positive. CTH workers estimated that the likelihood of their already being infected with COVID-19 to be 21.5 ± 24.7% as compared to the 15.3 ± 19.5% estimate of NCTH workers (*p* = 0.0001). 50% (545/1099) of the CTH workers and 51% (168/330) of the NCTH workers responded that the most important cause of their stress was a fear of infecting their families (*p* = 0.7).

**Conclusions:**

By multivariable analysis the creation of a NCTH during the COVID-19 pandemic was not found to be associated with a decrease in the number of hospital workers with clinical anxiety. Hospital worker support resources can be focused on the at-risk groups identified in this study.

## Background

The World Health Organization (WHO) declared the COVID-19 virus to be a public health emergency on January 20, 2020. Soon afterward, on March 11, the WHO re-classified the problem as a pandemic.

The WHO published an interim guidance on March 4, 2020 entitled “Health workers exposure risk assessment and management in the context of COVID-19 virus” [1]. A subsequent WHO interim guidance from March 19, 2020 [2], emphasized that the COVID-19 pandemic inevitably places health care workers at risk.

In view of the challenges of treating patients possibly infected with COVID-19 as well as those documented with the disease, exposed health care workers can be psychologically stressed [3–8]. A study from China reported depression in 50% and anxiety in 45% of nurses and physicians in the epicenter of the pandemic, the city of Wuhan, versus 7.2% in less affected regions of China [9]. Within the healthcare system it is the hospital which is the most affected by the COVID-19 pandemic.

COVID-19 is the second pandemic of the twenty-first century. The first was the influenza A/H1N1 virus infection, also known as swine flu in 2009. It differed from the COVID-19 pandemic in that the A/H1N1–09 virus survival time is greatly affected by seasonal temperatures, many older people already had immunity through exposure to similar viruses in the past and by the end of 2009 an effective vaccine was available. Hospital staff worries during that previous pandemic have been reported [10]. Twenty-one percent of the hospital staff were reported as having mild to moderate psychological distress, with the highest rate (23.9%) among nurses and the lowest rate (12.3%) among auxiliary personnel. The most frequent concern of staff was infection of family and friends and the health consequences of the disease, but only 6.6% of hospital workers restricted their social contacts. Around 3 billion doses of H1N1–09 vaccine were produced and many of them discarded due to low demand. During the SARS outbreak in 2003 in Toronto, 43% of the infected people were health care workers [11, 12]. Protective gear was required for health care workers and socialization in the hospital was restricted. The present COVID-19 pandemic is different in its epidemiology and in the fact that full population lock-downs were used in an attempt to control the pandemic.

During the first wave of the COVID-19 pandemic in Israel the Hadassah Medical Organization made an administrative decision to operate one of its two hospitals in Jerusalem as a COVID-19 treating hospital (CTH) and the other to be a non-COVID-19 treating hospital (NCTH), The NCTH continued to offer general medical services, doing elective procedures and surgery. A hospital by nature is a closely knit environment in which the efforts and coordination of all types of workers, both medical and non-medical are needed to provide good health care. During medical emergencies this interdependence is even more pronounced. The purpose of the present study was to assess the effect of the decision to create a CTH and a NCTH during the first wave of the COVID-19 pandemic on the level of clinical anxiety among all of the components of the workforce between the two hospitals. The findings can be informative for decision making during the current second and possible future waves of the pandemic. The authors hypothesized that CTH workers would have a higher percentage of clinical anxiety than NCTH workers.

## Methods

The Hadassah Medical Organization has two hospitals in Jerusalem. One hospital is located at Ein Kerem in the western part of the city and is an 800-bed level-3 hospital. The second hospital is located at Mt Scopus in the eastern part of the city and is a 300-bed level-2 hospital. During the height of the first wave of the COVID-19 pandemic, the Hadassah Medical Organization decided that the level-3 hospital would suspend all elective procedures and operations and function as a COVID-19 treating hospital (CTH). Services included dedicated COVID-19 wards for mild and moderate patients, ICU units for severe patients and a COVID-19 emergency room. The level-2 hospital was kept functioning as a non-COVID-19 hospital (NCTH), providing general medical services and continued to perform elective procedures and operations. While it had a separate emergency room for screening any patient suspected of having COVID-19, all patients diagnosed with COVID-19 were transferred to the CTH.

After receiving institutional review board approval (0281–20-HMO), a pilot was done to check that the electronic questionnaire was clear and that the automatic recording system worked properly. After necessary adjustments were made, a link to the electronic questionnaire was sent by hospital internal email and also to the mobile phones of each of the CTH and NCTH workers between April 27–30. Responses were accepted only until noon on May 1. Replies received afterwards were not included in the analysis because government plans to end some of the provisions of the COVID-19 lock-down were announced at that time. The questionnaire is anonymous and the details were automatically sent to an Excel spreadsheet (Microsoft Corp. Redmond, WA.) using Google Forms (Google Mountain View, CA).

The questionnaire has two parts. The first part is a survey of personal demographics and a questionnaire about specific issues related to COVID-19. The survey is presented in Table 1.

**Table 1:** The personal demographics questionnaire

1. Gender:	male / female / other
2. Age:	___
3. Who lives in your household?	spouse / parents / children / siblings / flat mate / alone / other
4. In which hospital do you work?	Ein Kerem / Mount Scopus / other
5. What is your position in the hospital?	senior doctor / resident / nursing staff / intern / lab worker / clerical staff / general services / technician / physical or occupational therapist / dietician / national service volunteer / nurse aid / volunteer / social worker or psychologist / other
6. In what department do you work?	______________________
7. Are you on a COVID-19 Department team?	yes / no / other
8. Are you currently working?	working in the hospital / COVID-19 home isolation / at home for other reason (maternity leave, other illness, on work leave without pay ect.)
9. The following are risk factors for COVID-19. Do you suffer from any of them?	diabetes / obesity / hypertension / chronic heart disease / chronic lung disease / smoker
10. Have you been in isolation because of the COVID-19?	yes / no
11. Have you have been tested for COVID-19?	yes / no
12. What were the COVID-19 test result?	positive /negative / results not received
13. Did you feel relieved after getting tested?	yes / maybe / no
13. Of all of the following, which is of the most concern?	getting COVID-19 / infecting family / giving corona to patients / my children are at home when I am in hospital / financial problems / professional burnout / other
14. Of the following, which would make the corona epidemic easier for you?	better protective gear / educational solution for my children / psychological support / group support / financial help / other
15. What percentage do you estimate that you already have gotten the corona virus?	______%

The second part of the questionnaire is a validated Hebrew translation of the 20-question portion of the State-Trait Anxiety Inventory for Adults (STAI-S) assessing anxiety state [13]. The S-Anxiety scale requires that the participant describe how he or she feels “now, at the present moment”. The scoring weight for the 10 anxiety present questions is: 1- Absolutely not; 2- A little; 3- Much; 4- Very Much. The scoring weight for the 10 anxiety absent questions is reversed. The total score varies from 20 to 80, and the higher the values, the greater the anxiety level. The questions composing the STAI-S are presented in Table 2. There are no published specific normative STAI-S values for hospital staff. Values are available for college students (36.47 ± 10.01 for males and 38.77 ± 11.90 for females), [13]. Bunevicius et al. [14], in their study of cardiac patients considered the cut-off value to represent clinical anxiety to be ≥45. When tested against other known measures for clinical anxiety they found that the STAI-S had a sensitivity of 89% and a specificity of 56%. The Bunevicius et al. [14] criteria were used in this study, with a score of ≥45 was considered to represent clinical anxiety [14].

**Table 2:** The questions composing the State-Trait Anxiety Inventory for Adults (STAI-S)

STAI-S	
Question Number	Question
1	I feel calm
2	I feel secure
3	I am tense
4	I feel strained
5	I feel at ease
6	I feel upset
7	I am presently worried about possible misfortune
8	I feel satisfied
9	I feel frightened
10	I feel comfortable
11	I feel self confident
12	I feel nervous
13	I am jittery
14	I feel indecisive
15	I am relaxed
16	I feel content
17	I am worried
18	I feel confused
19	I feel steady
20	I feel pleasant

### Statistical analysis

Statistical analysis was performed using the Statistical Analysis System (SAS Institute Inc., Cary, North Carolina, USA, version 9.4). Normally distributed interval data were compared across the groups, using the 2-tail Student’s t-test. Comparison of non parametric data was done using the Mann-Whitney *U* test. Nominal data were assessed with the chi-square test and Fischer’s exact test. Multivariable analysis to determine potential risk factors for increased STAI-S scores was performed using the generalized linear model (GLM) procedure. Variables measured by univariable analysis with *p* < 0.05 were entered into the model. In addition, stepwise multivariable logistic regression was performed to determine risk factors for clinical anxiety based on those with STAI-S scores ≥45. The association between risk factors and outcomes are presented as odds ratios (ORs) and 95% CLs after adjustment for confounders. Hospital, resident doctor, senior doctor, nurse, administrative staff, age ≥ 50, gender, presence of risk factors for COVID-19 and parents in the household were entered into the model. Data for employees who worked at both centers was excluded for any analysis that compared the CTH with the NCTH. To assess possible study sample size bias a comparison of major demographic variables of the surveyed study population and available total workforce data for each hospital was performed.

## Results

Complete questionnaires were received from 1570 of the 6528 hospital employees (24%). The mean total STAI-S score for all of the workers was 42.4 ± 11.8. 33.5% of the workers had STAT-S scores ≥45 indicating the presence of clinical anxiety. Table 3 presents the mean ± SD, median and interquartile range (IQR) and the percentage of those with STAI-S scores ≥45 according to worker categories. The highest anxiety scores were among dentists and resident doctors and the lowest among senior doctors.

**Table 3:** State-Trait Anxiety Inventory (STAI-S) scores according to major worker categories

Worker Category	No.	Mean ± SD	Median (IQR)	% STAI-S ≥ 45
Dentists	13	47.9 ± 12.9	49 (36–52)	53.8%
Resident doctors	117	46.3 ± 12.2	47 (37–56)	48.7%
Others	54	42.0 ± 11.9	41 (35–50)	38.9%
Nurses	487	44.2 ± 11.9	43 (35.5–53)	37.9%
Research staff	58	43.3 ± 11.9	45 (35–52)	37.9%
Office staff	234	42.4 ± 11.7	42 (33.5–52)	33.6%
Physician Assist.	10	37.5 ± 12.0	37 (27–46)	30.0%
Lab workers	93	42.4 ± 10.6	43 (34–50)	29.0%
Social workers/ psychologists	42	42.2 ± 9.7	41 (35–50)	28.5%
Non-physician clinicians	75	40.6 ± 11.0	39 (33.5–46.5)	28.0%
General service	62	40.2 ± 11.0	39 (33–49)	27.4%
Interns	18	38.4 ± 12.0	43 (27–47)	27.7%
Technicians	75	40.9 ± 11.3	40 (33–48)	25.3%
Senior doctors	220	38.0 ± 11.8	36 (28–46)	23.6%
Pharmacists	12	38.9 ± 7.2	41 (37–44)	8.3%
All staff	1570	42.4 ± 11.8	42 (34–51)	33.5%

By univariate analysis, overall workers at the CTH had a higher mean STAI-S score (43 ± 11.7) than workers at the NCTH (40.8 ± 11.8), [*p* = 0.005]. The percentage of overall CTH workers (35%) with STAI-S scores ≥45 was higher than the percentage of overall NTCH workers ((29%) who had STAI-S scores ≥45, but these differences were not supported by multivariable analysis. Differences in the STAI-S scores between the two hospitals for any of the individual worker categories were not statistically significant by univariate analysis.

The mean and ≥ 45 State-Trait Anxiety Inventory (STAI-S) scores of the hospital workers were further analyzed using univariable analysis according to the demographic data collected from the questionnaire. Table 4 presents these analyses according to major demographic groupings.

**Table 4:** Mean and ≥ 45 STAI-S scores of workers according to major demographic variables

Group	No.	Median STAI-S (IQR)	***P*** value	% STAI-S ≥ 45	***P*** value
CTH	1213	42 (34–52)	0.005	34.8%	0.04
NCTH	340	40 (31–49)		28.8%	
Males	442	38 (30–48)	0.0001	26.9%	0.0008
Females	1125	43 (35–52)		35.8%	
Medical risk factors	512	43 (34–50)	0.03	36.7%	0.006
No medical risk factors	1052	41 (33–50)		29.9%	
Tested for COVID-19	1082	42 (34–51)	0.13	34.6%	0.1
Not tested	480	41 (33–50)		30.6%	
Quarantined	201	42 (34–53)	0.55	32.3%	0.7
Not quarantined	1360	42 (34–51)		33.5%	
Age > 50	485	37.5 (29–47)	0.0001	23.2%	0.0001
Age < 50	1064	43 (35–53)		38.6%	
Senior doctors	220	36 (28–48)	0.0001	23.6%	0.0001
Resident doctors	120	46.5 (36–56)		48.3%	
Nurses	466	43 (35–53)	0.0002	38.0%	0.0007
Non-nurses	1088	41 (33–50)		29.5%	
Have children	929	42 (33–51)	0.3	33.1%	0.3
No children	708	43 (34–52)		30.7%	
Parent in household	162	45 (37–52)	0.02	42.0%	0.004
No parent in household	1475	42 (33–51)		30.9%	

Thirty-two point six percent of the workers had risk factors for COVID-19. Nine of the 1082 hospital workers who reported that they had taken a COVID-19 test stated that the test was positive. Administrative hospital data compiled at the time of the study showed that 38 workers had positive COVID-19 tests, 28 from the CTH and 10 from the NCTH. Workers at the CTH estimated that the likelihood of their already being infected with COVID-19 to be 21.5 ± 24.7%. This was significantly higher than the 15.3 ± 19.5% estimation of the NCTH workers (*p* = 0.0001). 43% (474/1093) of the CTH workers and 46.5% (138/297) of NCTH hospital workers responded that the most important stress reliever was better protective gear (*p* = 0.3). 17% (190/1093) of the CTH workers and 18% (54/297) of the NCTH workers responded that the most important stress reliever was a permanent arrangement for their children (*p* = 0.7). 50% (545/1099) of the CTH workers and 51% (168/330) of the NCTH workers responded that the most important cause of their stress was a fear of infecting their families (*p* = 0.7).

By multivariable analysis higher STAI-S scores were found to be associated with CTH hospital (*p* = 0.005), female gender (*p* = 0.001), age ≤ 50 (*p* = 0.001), those with risk factors for COVID-19 (*p* = 0.001), being a resident doctor (*p* = 0.001) and being a nurse (*p* = 0.001).

The results of stepwise multivariable regression analysis performed to identify risk factors for clinical anxiety as defined by STAI-S scores ≥45 is presented in Table 5.. Working in the COVID-19 treating hospital was not found to be a significant factor by this analysis.

**Table 5:** Risk factors for hospital staff clinical anxiety according to multivariable regression analysis

Variable	Odds Ratio (95% CI)	***P*** value
Resident doctor	2.13 (1.41–3.23)	0.0003
Age ≤ 50 years	2.08 (1.62–2.67)	<.0001
Nurse	1.29 (1.01–1.64)	0.0399
Female	1.63 (1.25–2.13)	0.0003
Having risk factors for COVID-19	1.51 (1.19–1.91)	0.0007

Demographic data for the total workforces of the CTH and NCTH were obtained from the Office of Manpower of the Hadassah Medical Organization and from published control reports. The surveyed population was not statistically different from the general population of each hospital for the percentage of nurses, senior doctors, resident doctors, and those age ≤ 50 years. There was a difference in the percentage of females in the CTH compared to the CTH survey population, but not for the NCTH. Table 6 presents this comparison data. We were unable to obtain risk factor data for COVID-19 for the total workforce.

**Table 6:** Comparison of the percentage of workers of the surveyed population and total hospital workforce data of each hospital according to demographic variables

Variable	Percentage of Worker Population					
	CTH			NCTH		
	Survey Population	Total Population	*P* value	Survey Population	Total Population	*P* value
Nurses	136 **(40%)**	479^a^ **(39.1%)**	0.41	349 **(28.8%)**	1859^b^ **(31.3%)**	0.08
Senior Physicians	45 **(13.2%)**	123^a^ **(10%)**	0.69	166 **(13.7%)**	717^b^ **(12.1%)**	0.12
Resident Physicians	25 **(7.4%)**	71^a^ **(5.8%)**	0.49	91 **(7.5%)**	540^b^ **(9.1%)**	0.07
Female	248 **(72.9%)**	1007^c^ **(70%)**	0.28	870 **(71.7%)**	3215^c^ **(63.2%)**	0.0001
Age ≤ 50	215 **(63.2%)**	977^c^ **(67.9%)**	0.1	861 **(71%)**	3623^c^ **(71.2%)**	0.88
Denominator	340	^a^1126 ^c^1439		1213	^b^5930 ^c^5089	

^a^Control Report Hadassah University Hospital Mt Scopus (06/06/2018). https://www.health.gov.il/PublicationsFiles/HadasaHZ_06062018.pdf

^b^Control Report Hadassah University Hospital Ein Kerem (11/04/2018). https://www.health.gov.il/PublicationsFiles/HADASA_EC_11042018.pdf

^c^Office of Manpower Hadassah Medical Organization

## Discussion

The hypothesis that a higher percentage of CTH workers would have clinical anxiety than NCTH workers was not found to be true in this study. While the univariate analysis suggests that a higher percentage of workers in the CTH than in the NCTH had clinical anxiety according to STAI-S scores ≥45 [14], this difference was not supported by multivariable analysis. When specific categories of workers were analyzed for the effect of working in the two hospitals on their mean anxiety and the percentage with clinical anxiety, no differences were found. This was also true for resident doctors and nurses who in the CTH were on the frontline of COVID-19 treatment.

Why the creation of a NCTH did not result in a lower level of worker clinical anxiety can have several possible explanations, some of which are intrinsic and some extrinsic to the hospital. First of all, the creation of a NCTH might not have been equated by the hospital staff to mean that it was coronavirus free. The possibility of NCTH staff contact with patients carrying the coronavirus in the emergency room existed. There also was the possibility of exposure to patients who were admitted to the hospital with undiagnosed COVID-19. Another explanation may be that the anxiety caused by the population lock-down was a more dominant factor in causing hospital worker anxiety than factors intrinsic to their hospital workplace. The results could also have been affected by sample bias due to the survey low response rate.

To check for possible sample bias a comparison was done between the surveyed population and total workforce data from each hospital. The variables nurses, senior doctors, resident doctors, those of age ≤ 50 years and female gender were assessed. Statistical analysis showed that the surveyed population was not different from the general population of each hospital for the percentage of nurses, senior doctors, resident doctors, and those age ≤ 50 years. There was a difference in the percentage of females in the CTH compared to the CTH survey population, but not for the NCTH. This analysis indicates that the likelihood of study sample bias is low.

Lai et al. [9], reported on the mental health outcomes among nurses and physicians exposed to COVID-19 in the hospitals of Wuhan China during the pandemic. The nurses and physicians in Wuhan, who were part of the Lai et al. [9] study, were in an environment similar to that of the hospital workers in the current study in that both were working in an environment of a COVID-19 pandemic lock-down. In their study, they found by multivariable analysis that frontline health care workers engaged in direct diagnosis, treatment and care of patients with COVID-19 had a higher risk for symptoms of anxiety, insomnia and distress. In the current study using multivariable analysis, there was no significant difference found in the percentage of workers with clinical anxiety in the CTH, which was frontline and workers in the NCTH which was not frontline. Among those on the front-lines Lai et al. [9] 50.4% suffered from depression, 44.6% from anxiety and 71.5 from insomnia. Similar levels of those with clinical anxiety were found in this study, with 49% of resident doctors and nurses having clinical anxiety. That the senior doctors in this study were on the opposite end of the clinical anxiety spectrum with 24% effected, may reflect their experience in confronting crises medical crises and/or less involvement in direct patient care. This study differs from the Chinese study [9] in that it was done in a country with a different governmental system and where the COVID-19 pandemic was considered at the time of the study to be under control. At the time when this study was performed, less than 200 people had died and 16,000 had been diagnosed in a country with a population of 9.1 million.

The study of Giulia et al. [10] of psychological distress in a single Greek tertiary teaching hospital during the swine flu pandemic took place in a social system closer to the present study than in the Lai et al. study [9]. They found that the degree of anxiety and perceived risk of infection were both moderately high among health care workers. There however were no reported deaths in their hospital from swine flu during their study.” Two years after the SARS outbreak in Toronto, Maunder et al. [15] reported that health care workers who treated SARS patients had elevated rates of signs of chronic stress than workers who did not treat SARS patients.

This study identified hospital workers at risk for clinical anxiety in the COVID-19 pandemic. By multivariable analysis being a medical resident, age ≤ 50 years, being a nurse, female gender and workers with risk factors for COVID-19 were all found to be risk factors and odds ratios were calculated for each. This information is important because it can help focus administrative support to high risk groups among hospital workers during future waves of COVID-19 or other future pandemics.

While 69% of the hospital workers in this study had been tested for COVID-19, only nine tested positive for COVID-19. The low rate of infection (6.7/1000 workers) based on the data from the questionnaire is similar to the rate (5.4/1000 workers) calculated from the data compiled by medical organization administration. It reflects the fact that the lesson of having good protective gear available for the hospital staff was learned from the counties effected earlier in the pandemic. Forty-four percent of the workers in this study indicated that having good protective gear relieved their stress.

The workers in the CTH estimated that they had a 21% chance of having already contracted COVID-19 as opposed to the 15% estimate of NCTH workers (*p* = 0.0001). This is in spite of the high number of workers who had polymerase chain reaction tests. It reflects the knowledge of the hospital workers that having a single negative polymerase chain COVID-19 test does not mean conclusively that a person does not, or did not have COVID-19.

A weakness of this study is the relatively low percentage of responders to the questionnaire. This can result in study bias and possibly over estimate anxiety. The low response rate is a function of the fact that the survey was not administered, that data collection was limited to only a 4 day period and that most of the hospital workers were working extra long shifts and opening an electronic message and responding to a questionnaire may not have been a priority for them. In spite of the low response rate, analysis showed that surveyed population was similar to the total workforce population in major demographics. Given these conditions, we believe that the study offers a valuable snapshot assessment of the anxiety and attitudes of hospital workers during the population lock-down stage of the COVID-19 pandemic. It is the largest study cohort to date measuring hospital worker anxiety during the COVID-19 pandemic. It is the only study that compares hospital worker anxiety in COVID-19 treating and non-treating hospitals within the same medical system in the same city. It also surveys the entire hospital staff and not just nurses and doctors.

The current study was done in a COVID-19 pandemic environment, with a population lock-down present. The study hypothesis that CTH workers would have a higher percentage of clinical anxiety than NCTH workers was not found to be true. While not having the hypothesized effect, the administrative designation and operation of a hospital as a non-COVID-treating center allowed it to provide full and needed medical services. The greatest hospital worker concerns found in this study were having good protective gear and not infecting their family. In spite of having an ample supply of good protective gear and being in a country with early and effective national management of the COVID-19 pandemic, one third of the workers in both hospitals had clinical anxiety. This study identified both risk factors for clinical anxiety among hospital workers during the first wave of the COVID-19 pandemic as well as those workers most susceptible. Sustained staff clinical anxiety can have a detrimental effect on worker health and performance. The study findings can be used in decision making to help develop and focus supportive efforts in the current second wave of the pandemic and in possible future waves.

The hospital staff concerns identified in this study can potentially be mitigated by practical steps. One way is for hospitals to provide a safe as possible working environment. This means enforcement of proper patient mask wearing and social distancing between patients and staff. Social distancing should be practiced within the staff as well. Whenever possible staff personal meetings should be conducted remotely. COVID-19 testing should be readily available with a minimum of administrative hassle for both patients and staff. Scheduled routine testing of staff might also serve to decrease anxiety. Hospital management should target, monitor and offer support to high risk worker groups using remote meetings.

Another way to help address the concerns of the hospital staff is to minimize uncertainty by providing regular detailed hospital COVID-19 information at two levels. One level is specific to the staff’s own hospital and the other is country wide data. The staff should not learn of their hospitals COVID-19 status by hearsay. Work guidelines for hospital workers who have co-morbidities should be published. Online COVID-19 educational courses should be available for nurses, residents and senior doctors who do not ordinarily take care of internal medicine cases to prepare them if needed to treat COVID-19 patients. Sufficient reserve manpower also needs to be available to replace hospital workers who display high levels of clinical anxiety.

## Data Availability

The datasets used and/or analyzed during the current study are available from the corresponding author on reasonable request.
